# A Rare Manifestation of Uveitis-glaucoma-hyphema Syndrome

**DOI:** 10.5005/jp-journals-10008-1205

**Published:** 2016-08-05

**Authors:** David Cordeiro Sousa, Inês Leal, Mun Yueh Faria, Luís Abegão Pinto

**Affiliations:** 1 Resident, Department of Ophthalmology, Hospital de Santa Maria, Lisbon Academic Medical Center; Centro de Estudos Ciências da Visão Faculty of Medicine University of Lisbon, Lisboa, Portugal; 2 Resident, Department of Ophthalmology, Hospital de Santa Maria, Lisbon Academic Medical Center; Centro de Estudos Ciências da Visão Faculty of Medicine University of Lisbon, Lisboa, Portugal; 3 Resident, Department of Ophthalmology, Hospital de Santa Maria, Lisbon Academic Medical Center; Centro de Estudos Ciências da Visão Faculty of Medicine University of Lisbon, Lisboa, Portugal; 4 Assistant ProfessorDepartment of Ophthalmology, Hospital de Santa Maria, Lisbon Academic Medical Center; Centro de Estudos Ciências da Visão Faculty of Medicine University of Lisbon, Lisboa, Portugal

**Keywords:** Cataract surgery, In-the-bag intraocular lens spontaneous dislocation, Uveitis-glaucoma-hyphema syndrome.

## Abstract

**Aims:** To report a case of a patient who developed uveitis-glaucoma-hyphema (UGH) syndrome after an uneventful cataract surgery and to discuss risk factors, diagnostic challenges, management options, and clinical implications.

**Background:** Uveitis-glaucoma-hyphema syndrome is a rare but potentially serious cataract surgery complication. Clinical manifestations include increased intraocular pressure (IOP), anterior chamber inflammation, and recurrent hyphema or microhyphema. Uveitis-glaucoma-hyphema Plus syndrome also includes accompanying vitreous hemorrhage. Although classically associated with rigid anterior chamber intraocular lenses (lOLs), cases of malpositioning and subluxated posterior chamber lOLs have also been described as possible triggers.

**Case description:** We report a case of a 70-year-old Caucasian man who developed UGH Plus syndrome after an uneventful cataract surgery with an lOL implanted in the capsular bag. During postoperative follow-up, persistent intraocular inflammation, increased IOP, hyphema, and vitreous hemorrhage were consistent with this diagnosis. Slit-lamp examination demonstrated progressive localized iris atrophy, compatible with chafing of the posterior iris by the IOL haptic as the trigger for UGH syndrome. A pars plana vitrectomy was performed and a retropupillary intraocular lens was implanted. No further complications occurred during follow-up.

**Conclusion and clinical significance:** Given the increasing prevalence of single-piece lOLs implanted in the capsular bag, it is important to recognize UGH syndrome as a rare but potentially serious complication.

**How to cite this article:** Sousa DC, Leal I, Faria MY, Pinto LA. A Rare Manifestation of Uveitis-glaucoma-hyphema Syndrome. J Curr Glaucoma Pract 2016;10(2):76-78.

## CASE REPORT

### Background

Worldwide, the number of single-piece intraocular lenses (IOLs) implanted in the capsular bag is growing.^1,2^ Among potential postoperative complications, one possible but seldom described is the uveitis-glaucoma-hyphema syndrome (UGH). Uveitis-Glaucoma-Hyphema syndrome or Ellingson’s syndrome was first described in 1978, and it is known to be caused by mechanical iris trauma, being classically associated with rigid anterior chamber IOLs.^3,4^ However, posterior chamber IOLs have been increasingly recognized as a cause of UGH syndrome due to poor fit, malpositioning, and/or subluxation of lens/capsular bag complex.^3,5-9^ The affected eye manifests with increased intraocular pressure (IOP), anterior chamber inflammation, and recurrent hyphema or microhyphema.^4,10,11^

We report a case of a 70-year-old Caucasian man who developed UGH syndrome after an uneventful cataract surgery.

### Case Description

**Fig. 1:**
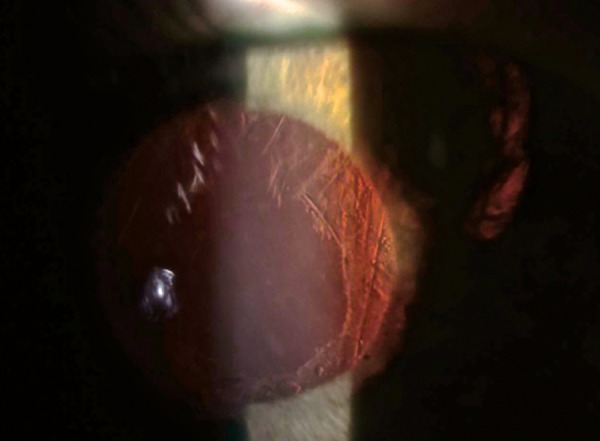
An haptic-like transillumation defect was identified due to chafing of the posterior by the dislocated intraocular lens

**Fig. 2:**
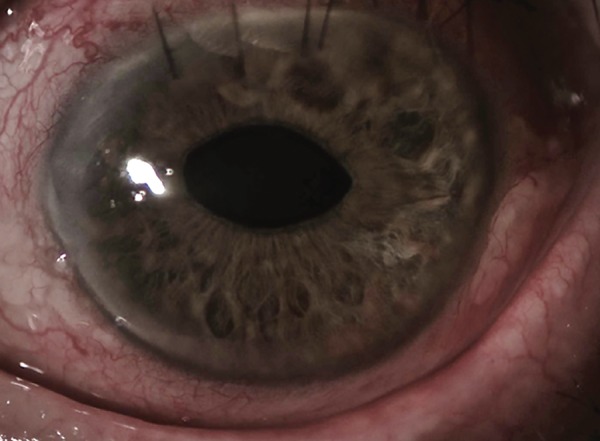
Postoperative photography of the anterior segment. A peaked pupil is noted in relation to the posterior Verisyse^®^ iris-claw, increased due to iris atrophy

**Fig. 3:**
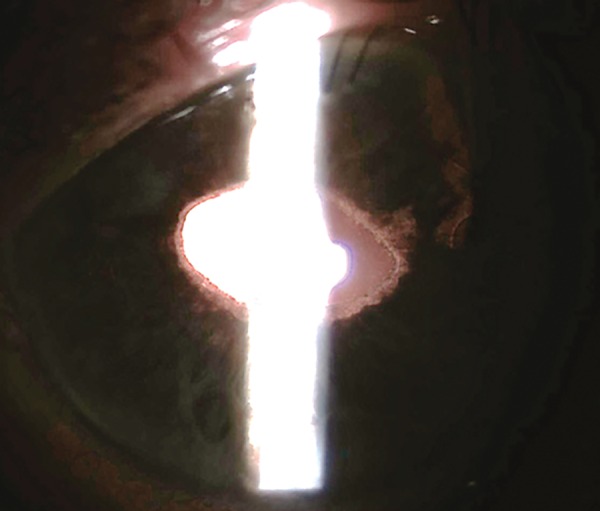
Postoperative aspect of the iris transillumination defect

A 70-year-old Caucasian man first presented to our Ophthalmology Department in 2010 for elective cataract surgery to his right eye. His medical history was unremarkable regarding systemic diseases and ophthalmolog-ical conditions. Slit-lamp examination revealed bilateral pseudoexfoliation syndrome (PXS) and a visually significant cataract in his right eye. Increased intraocular pressure was 28 mm Hg OU and gonioscopy revealed a bilateral grade IV (Schaffer) angle. Fundoscopy disclosed an asymmetric cup-to-disk ratio (0.7 *vs* 0.4), increased in the right eye. At this time, visual field testing and retinal nerve fiber layer analysis were within normal values. Cataract surgery was uneventful and a monofocal single-piece intraocular lens was implanted in the capsular bag. He remained under medical treatment (topical prosta-glandin analog) bilaterally and scheduled for routine follow-up in the glaucoma department. Three years later, following the same operative protocol (including IOL type), a similarly uneventful cataract surgery was performed on his left eye. One month after surgery, his BCVA (best corrected visual acuity) was 20/20 OD and 20/25 OS, the IOL was described as centered in-the-bag and IOP was 17 mm Hg OU under treatment. One year later, the patient complained of blurry vision and persistent discomfort in the left eye. Best corrected visual acuity was 20/25 OD and 20/80 OS. Slit-lamp examination disclosed anterior chamber white cells, IOP was 24 mm Hg OD and 32 mm Hg OS, and gonioscopy was repeated (detailing an asymmetry in pigment between the two eyes, but with IV Schaffer bilaterally with no other abnormalities). A few days later, the patient complained of reduced visual acuity in the left eye. A total hyphema was identified and B-scan ultrasound of the left eye revealed a vitreous hemorrhage with no retinal detachment. The patient underwent a pars plana vitrectomy during which no apparent cause for the bleeding was detected. Postoperatively, a slit-lamp examination revealed anterior chamber inflammation an upward arciform peripheral transillumination iris defect (Fig. 1), in possible relation to an IOL malpositioning or subluxation. Then, the IOL was explanted and a retro-pupillary Verisyse^®^ IOL was put in place (Fig. 2). After 2 months of follow-up (Fig. 3), IOL is stable, the iris defect is still identifiable, but no anterior chamber inflammation or pigment dispersion was found. Increased intraocular pressure was 16 mm Hg OD and 18 mm Hg OS under the same topical monotherapy.

## DISCUSSION

In the described case, a UGH syndrome developed following uneventful cataract surgery. Of note, the fellow eye had been operated 3 years before with no complications.

In a recent paper, Zhang et al^5^ proposed two mechanism for in-the-bag UGH syndrome: (i) Subclinical phacodonesis of the haptic-capsule complex in a patient with PXS resulting in chafing of the posterior iris and (ii) focal anterior capsule fibrosis around the haptic, creating a mechanical contact with the posterior iris. In this line of thought, we believe the observed localized iris atrophy to be related with a dislocation of the implanted IOL with prolonged haptic-induced trauma of the posterior iris (Fig. 1). This concept is supported by the apparent sudden aggravation of the condition after a gonioscopy examination, during which the pressure and/or suction effect caused by the two-mirror goniolens may have been the eliciting factor due to vessel damage of his very delicate and thin iris.

Although rare, late in-the-bag single-piece IOL dislocation is being increasingly recognized, with incidence rates varying from 0.1 to 3.0% of postoperative cataract patients.^12-14^ Identified risk factors for this condition include PXS, connective tissue disorders, high myopia, and patients who underwent vitreoretinal surgery.^15^ The incidence of ocular surgery meant to correct a dislocated IOL ranges from 0.03 to 0.28% .^14,16^ Since the pseudophakic population is growing as a consequence of increased lifespan and better and safer surgical technology, one may expect that the number of in-the-bag IOL dislocation may become a common issue in the future. As previously described, ultrasound biomicroscopy (UBM) would have been useful in confirming the diagnosis preoperatively, by determining the relative position of the IOL haptic and the iris.^17^

In these cases, topical steroid medication and aggressive IOP-lowering treatment are usually of limited use as they do not address the stimulus for inflammation.^3,7^ However, not all patients are suitable for IOL exchange and the advantages of removing the aggravating factors should be weighed against risk of a new intraocular surgery, potential corneal damage and decreased visual acuity.^6^ The case described presented with UGH syndrome and vitreous hemorrhage, known as UGH Plus syndrome, is an even rarer cataract surgery complica-tion.^18^ The occurrence of a vitreous hemorrhage in this case raises the possibility of weak zonules as a trigger for this situation, thus allowing for communication between the posterior chamber as the vitreous. After discussing the case with vitreo-retinal department and hearing patient’s preferences, a pars plana vitrectomy was performed and a retropupillary Verisyse^®^ IOL implantation was considered the best option in this specific case. No further complications occurred during follow-up.

## CONCLUSION AND CLINICAL SIGNIFICANCE

A careful slit-lamp postoperative examination is crucial to confirm normal positioning of the IOL optic and haptics in the capsular bag. Also, since in-the-bag IOL may sometimes cause pigment dispersion and UGH syndrome, it is of paramount importance to look for signs of posterior iris rubbing, namely, pigment dispersion, anterior chamber inflammation, and IOP elevation. A personalized approach should be adopted for each case, taking into account the presence of potential risk factors (e.g., PXS), relative contraindications for a new intraocular surgery, and the patient’s preferences.

Given the increasing prevalence of implanted single-piece IOL in the capsular bag, it is important to recognize UGH as a rare complication but associated with potentially serious morbidity.
